# Socio-Behavioral Risk Factors Associated with Cryptosporidiosis in HIV/AIDS Patients Visiting the HIV Referral Clinic at Cape Coast Teaching Hospital, Ghana

**DOI:** 10.2174/1874613601812010106

**Published:** 2018-09-12

**Authors:** Yeboah K. Opoku, Johnson N. Boampong, Irene Ayi, Godwin Kwakye-Nuako, Dorcas Obiri-Yeboah, Harriet Koranteng, George Ghartey-Kwansah, Kwame K. Asare

**Affiliations:** 1 Department of Biomedical Sciences, School of Allied Health Sciences, Collage of Health and Allied Science, University of Cape Coast, Cape Coast, Ghana; 2 Biopharmaceutical Laboratory, College of Life Sciences, Northeast Agricultural University, Harbin 150030, China; 3 Department of Parasitology, Noguchi Memorial Institute for Medical Research, College of Health Sciences, University of Ghana, P. O. Box LG 581, Legon, Accra, Ghana; 4 School of Medical Sciences, University of Cape Coast, Cape Coast, Ghana; 5 Jiamusi University No. 148, Xuefu Road, Jiamusi, Heilongjiang, China; 6 Laboratory of Cell Biology, Genetics and Developmental Biology, College of Life Sciences, Shaanxi Normal University, Xi'an 710062, China; 7 Department of Protozoology, Institute of Tropical Medicine (NEKKEN), Nagasaki University Sakamoto 1-12-4, Nagasaki 852-8523, Japan; 8 Leading Program, Graduate School of Biomedical Sciences, Nagasaki University, Nagasaki, Japan

**Keywords:** *Cryptosporidium* oocyst, HIV/AIDS, CD4+, Risk factors, Sachet water, Chronic diarrhea, HIV referral clinic, Cape Coast, Ghana

## Abstract

**Objective:**

To identify the socio-behavioral risk factors associated with cryptosporidiosis among HIV/AIDS patients with chronic diarrhea symptoms visiting the HIV referral clinic at Cape Coast Teaching Hospital, Ghana.

**Methods:**

A cross-sectional study was conducted among 50 HIV/AIDS patients with recurrent diarrhea. Questionnaires were administered to collect social and behavioral risk factors associated with *Cryptosporidium* and other opportunistic protozoan parasitic infections in HIV patients. Stool samples were collected for the diagnosis of enteric protozoan pathogens using modified Ziehl-Neelsen and acid-fast staining methods. CD4^+^ cells counts of study subjects were obtained from patients clinical records. The data obtained were analyzed using Pearson chi-square and multivariate-adjusted statistics tool on SPSS 16 for Windows.

**Results:**

Twenty-seven (54%) of the subjects were infected with enteric protozoan pathogens. The prevalences of *Cryptosporidium*, *Cyclospora* and *Microsporidium* infections were 46%, 32% and 16%, respectively. *Cryptosporidium* infection was significantly associated with drinking water (×^2^=13.528, p<0.001), *Cyclospora* was associated with the type of drinking water (×^2^=14.931, p<0.001) and toilet facilities used by the study subjects (×^2^=12.463, p<0.01), whiles *Microsporidium* infection was associated with hand washing behavior (×^2^=12.463, p<0.01). Enteric protozoans were frequently encountered among subjects with CD4+ T-cell count <200 cells/mm^3^. However, coinfection of *Cyclospora spp* & *Cryptosporidium* spp was not observed in CD4^+^ cell count <200 and >500 cells/mm^3.^ Multivariate analysis showed that the risk factor for *Cryptosporidium* infection among HIV/AIDS patients was the source of drinking water (pipe borne water 76.2% prevalence: sachet water 25%; OR=0.10, 95%CI: 0.03-0.39, p<0.001).

**Conclusion:**

We report the risk factor for exposure of *Cryptosporidium* infection among HIV/AIDS patients for the first time in Ghana. The contamination of drinking water by protozoan parasites should be a public health concern. These results provide the stepping block to understand the transmission dynamics of *Cryptosporidium* and other opportunistic pathogens in HIV/AIDS infected patients in Ghana.

## 1 INTRODUCTION

Diarrhea related morbidity and mortality among HIV/AIDS patients are still a clinical challenge to both clinicians and affected individuals [1, 2]. This challenge emanates from the non-specificity of the etiological agents of chronic diarrhea in HIV/AIDs infection [3]. Chronic diarrhea caused by opportunistic enteric pathogens is increasingly observed in HIV/AIDS patients with depleted CD4^+^ T lymphocytes cells in Ghana [4]. Cryptosporidiosis, chronic diarrhea caused by *Cryptosporidium* infection, is one of the life-threatening clinical conditions affecting HIV/AIDS patients [5, 6]. Although highly active antiretroviral therapy (HAART) has shown to increase CD4^+^ cell levels among HIV/AIDS patients, cryptosporidiosis still remains high in these immunocompromised individuals [7]. In Ghana, the prevalence of cryptosporidiosis ranges from 12% to 28.6% among HIV/AIDS patients [8, 9].

Though there are increasing reports on the prevalence of cryptosporidiosis and its association with depleted CD4+ cells among HIV patients in Ghana, little is known about its transmission risk factors [10]. The only study which attempted to establish the socio-behavioral risk factors and transmission patterns of *Cryptosporidium* infection among HIV patients could not establish any significance between them [9]. Several factors have been associated with *Cryptosporidium* infection in HIV/AIDS infections [11-14]. A number of research findings have shown an association between *Cryptosporidium* and consumption of untreated water [15, 16]. The detection of *Cryptosporidium* spp. from potable water sources in Ghana suggested that improper treatment of drinking water could be a key transmission source for *Cryptosporidium* infection [17, 18].

The transmission of *Cryptosporidium* is very complex especially in poor sanitation environments [19]. *Cryptosporidium spp*, *Cyclospora spp*, *Microsporidium spp*, and other microorganisms have been detected in plastic packaged drinking waters “sachet water” within Ghana [20, 21]. Cryptosporidiosis is self-limiting diarrhea in immunocompetent individuals but becomes severe, chronic and life-threatening in children under five years and immunocompromised individuals especially those with profound malnutrition or wasting [22]. Host immunity which plays a protective role against severe cryptosporidiosis is boosted upon exposure to *Cryptosporidium* infection [23]. In children under five years, clinical severity of cryptosporidiosis ameliorates with therapy from the onset of the disease [24]. The boosted acquired immunity protect such children against subsequent severe cryptosporidiosis upon infection. In HIV infections, the priming of CD8^+^ cell to kill CD4^+^ cells causes an imbalance between Th1 and Th2 cytokines and as a result predisposes the host small intestine to damage by inflammation [25]. *Cryptosporidium* infection augments the imbalance between Th1 and Th2 cytokines in an immunocompromised position leading to prolong chronic life-threatening diarrhea [26, 27].

In our previous report, we showed that HIV/AIDS patients with CD4+ cell count below 200 cells/mm^3^ were frequently predisposed to cryptosporidiosis [28]. Other researchers have also reported similar findings in other parts of Ghana [8, 23]. However, a few studies from Ghana have tried to identify an association between socio-behavioral risk factors and cryptosporidiosis among HIV infected patients. The zoonotic exposure of *Cryptosporidium* spp results in continuous circulation and transmission cycle in poor sanitation environments [29]. The identification of common exposure sources of *Cryptosporidium* infection among HIV patients could assist clinicians to offer the needed advice to this group of patients on how to reduce the risk of infection.

This study focuses on identifying social and behavioral factors that influence cryptosporidiosis infection in HIV patients visiting the HIV referral clinic in the Central region. This report has public health significance on the transmission of cryptosporidiosis in the immunocompromised host. The results being the first report to understand the transmission dynamics in *Cryptosporidium* spp and other opportunistic protozoan parasites in HIV/AIDS infection in Ghana could lead to policy formulation and rational approaches to control cryptosporidiosis and other opportunistic pathogens in HIV/AIDS infection.

## 2 MATERIAL AND METHODS

### 2.1 Study Site

A cross-sectional study was conducted at the HIV referral clinic in Cape Coast, Ghana. The clinic serves as a referral clinic for all the HIV clinics within seventeen districts in the region. The HIV referral clinic is located within the Cape Coast Teaching hospital situated at Cape Coast, the capital city of the region. The Central region is a governmental administrative area of size 9,826 km^2^ inhabited by about 2.2 million people. According to the 2016 HIV sentinel summary report, the HIV prevalence in the Central region stands at 1.8%.

### 2.2 Ethical Approval

Ethical clearance was obtained from the Ghana Health Service Ethical Review Committee on Research Involving Human Subjects (ERCRIHS), certificate number GHS-ERC: 24/01/12. Permission to interact and collect research samples from HIV infected clients assessing the HIV referral clinic was obtained from the administration of the hospital.

### 2.3 Selection of Study Subjects

The purpose of the study was explained to the potential subjects and those who accepted to take part in this research were enrolled in the study. The results of the participants were linked to their clinical data for prompt treatment if necessary with their consent and also their confidentiality assured that the data would not be released outside the health facility. In all fifty (50) subjects participated in the study from May 2012 to April 2013. All the participants were HIV/ AIDS patients who had been diagnosed with at least three days of recurrent diarrheal condition at the referral clinic. Unique identification (ID) numbers were given to each of the study subjects after accepting and signing the informed consent form. These unique ID numbers were also given to the stool samples and the administered questionnaires. These procedures boosted the confidence of the participants to effectively contribute to the study since participant names were not recorded in the research registrar. However, we were able to link the ID numbers to the patient’s clinical folder, which made it easier to report any enteric pathogenic infections to the clinician in charge of the referral clinic for prompt treatment.

### 2.4 Questionnaire Administration and Sample Collection

Structured and semi-structured questionnaires with translations in local dialects were administered to the subjects enrolled in the study. Guardians and parents of subjects below 12 years of age were assisted in answering the questionnaires. The questionnaires were used to obtain socio-demographic data such as age, sex, residence, educational status and possible behavioral risk factors associated with the transmission of enteric protozoan parasites such as hand and fruit washing practices, type of toilet facility, and source of drinking water. CD4 ^+^ cell counts of the participants were also obtained from their clinical folders.

Through the use of motion pictures and illustrations, all the fifty (50) participants were educated on how to collect stool samples without contamination. A sterile plastic container (labelled with subjects unique ID to match the questionnaire and clinical folder) with a tight-fitting lid which contained an applicator to scoop the stool samples were given to each subject. The stool samples collected at the clinic were stored at 4^o^C for a maximum of 12 hours and were transported on ice to the laboratory of the Department of Biomedical and Forensic Sciences, University of Cape Coast for pathogenic diagnostics and analysis.

### 2.5 Analysis of Stool Samples

The stool samples were immediately processed on arrival by wet mount, formalin-ethyl acetate sedimentation and microscopic observations were made to identify possible enteric pathogens in the samples. Four thin slides were made from each stool, air dried and fixed in absolute ethanol. The slides were then stained using Modified Ziehl-Neelsen and other acid-fast staining methods followed by microscopic identification of protozoan parasites [28].

### 2.6 Statistical Analysis

The data collected from both questionnaires and stool samples were keyed into Microsoft Excel 2007 (Microsoft Corporation) and analyzed using SPSS statistical Software for Windows version 16 (SPSS Inc.). The prevalence of each protozoan pathogenic infection was estimated using proportions. All statistical associations in this study were tested using Pearson chi-square test; p-value ≤ 0.05 was considered statistically significant. Multivariate-adjusted statistics at 95% confidence interval (CI) was used as robust statistics to confirm risk factors associated with the exposure of HIV/AIDS patients to opportunistic pathogens.

## 3 RESULTS

Fifty (50) subjects with recurrent diarrhea attending HIV/AIDS clinic at the Cape Coast teaching hospital, Cape Coast were enrolled into the study. All the subjects provided stool samples and answered the questionnaires designed to investigate the possible risk factors for *Cryptosporidium* infection among HIV/AIDS patients and their CD4^+^ data were also obtained from their clinical records. The study subjects consisted of 14 (28%) males and 36 (72%) females. Twenty-seven (54%) of the enrolled subjects had enteric protozoans in their stool samples.

Three protozoan parasites *Cryptosporidium* (46%), *Cyclospora* (32%) and *Microsporidium spp* (16%) were identified from the stool samples. These protozoan parasites were stratified into tables with social demographic and behavioral characteristics to identify the risk factors for cryptosporidiosis and other protozoan infections among the study subjects. Among the subjects, 6 (42.9%) out of 14 males and 10 (27.8%) out of 36 females had *Cyclospora* infections (×^2^=1.053, p>0.05); 8 (57.1%) of the males and 15 (41.7%) of the females had *Cryptosporidium* infections (×^2^=0.972, p>0.05) whilst 4 (28.6%) and 4 (11.1%) had *Microsporidium* infections in males and females respectively (×^2^=2.286, p>0.05). There was no significant differences among the age categories for *Cyclospora* (×^2^=8.323, p>0.05), *Cryptosporidium* (×^2^=2.287, p>0.05) *and Microsporidium* (×^2^=4.772, p>0.05); for residence, *Cyclospora* (×^2^=0.006, p>0.05), *Cryptosporidium* (×^2^=2.061, p>0.05) *and Microsporidium* (×^2^=0.214, p>0.05) (Table **1**). However, *Cyclospora* infections were significantly associated with the source of drinking water (×^2^=14.931, p<0.001) and the type of toilet facility used by the participants (×^2^=12.463, p<0.01), *Cryptosporidium* infection among the subjects in a similar trend was also associated with the source of drinking water (×^2^=13.528, p<0.001) whereas *Microsporidium* infection was associated with hand washing behavior of the subjects (×^2^=12.463, p<0.01). There was no significant association among the fruit washing behavior for *Cyclospora* (×^2^=0.146, p>0.05), *Cryptosporidium* (×^2^=1.567, p>0.05), and *Microsporidium* (×^2^=4.352, p>0.05) (Table **2**).

The frequencies of coinfection of enteric protozoans among the study subjects were classified as triple infection (*Cyclospora, Cryptosporidium and Microsporidium*) with a prevalence of 2 (14.3%) and 3 (8.3%) for males and females respectively; double infections of *Microsporidium* & *Cyclospora* were 2 (14.3%) and 1 (2.8%), and *Cyclospora* & *Cryptosporidium* were 1 (7.1%) and 5 (5.6%) for male and female subjects respectively; for single infections, the frequencies of *Cyclospora* infection were 1 (7.1%) and 1 (2.8%) whereas *Cryptosporidium* infection was 5 (35.7%) and 10 (27.8%) among male and female subjects respectively(Table **3**).

The distribution of enteric protozoan infections was grouped with respect to CD4^+^ T-lymphocyte counts. In all, patients with CD4+ cell count lower than 200 cells/mm^3^ had 61.5% prevalence of enteric protozoans; 2 (12.5%) had triple infections, 1 (6.3%) of *Microsporidium* & *Cyclospora* double infection, 1 (6.3%) of *Cyclospora* and 7 (43.8%) of *Cryptosporidium* as single infections whilst 5 (31.3%) of the subjects in this category had no enteric protozoan infections. Triple infection 1 (4.5%), double infections 1 (4.5%) and 3 (13.6%) for *Microsporidium* & *Cyclospora* and *Cyclospora* & *Cryptosporidium* infections respectively, and no single *Cyclospora* infection was observed among patients with CD4+ cell count between 200-499 cells/mm^3^ (Table **4**).

The risk factors associated with *Cryptosporidium* and other enteric protozoan infections among the study subjects were robustly analyzed using multivariate-adjusted statistics. The significant risk factor for *Cryptosporidium* infection among HIV/AIDS patients with recurrent diarrhea in this study was the source of drinking water [sachet water (OR=0.10, 95%CI: 0.03-0.39, p<0.001) and borehole water (OR=0.11, 95%CI: 0.00-3.14, p=0.198) with pipe borne water as a reference]. Similarly, *Cyclospora* infection also had a source of drinking water as a risk factor [sachet water (OR=0.07, 95%CI: 0.02-0.33, p<0.001) and borehole water (OR=0.02, 95%CI: 0.01-5.77, p=0.356) with pipe borne water as a reference]. The kind of toilet facility used by subjects was observed to be a risk factor for *Cyclospora* infection by Pearson chi-square statistics (p<0.01). On the contrary, multivariate analysis showed no significant association with the kind of toilet facility used by study subjects [public toilet (OR=0.92, 95%CI: 0.22-3.83, p=0.905), household toilet (OR=0.33, 95%CI: 0.01-8.63, p=0.508), and open defecation (OR=0.44, 95%CI: 0.04-5.40, p=0.512) with personal water closet as a reference]. Hand washing behavior (p<0.05) was identified as a risk factor for *Microsporidium* infection among the study subjects. Multivariate analysis showed that study subjects who never or rarely wash their hands (OR=20.45, 95%CI: 0.93-449.12, p=0.05) were more prone to *Microsporidium* infection compared to the rest of the subjects enrolled into the study (Table **5**).

## 4 DISCUSSION

Gastroenteritis triggered by *Cryptosporidium* infection causes prolonged or chronic diarrhea which leads to malnutrition and wasting, a life-threatening condition in HIV/AIDS infections [27, 30, 31]. A substantial number of chronic diarrhea in HIV infection is as a result of cryptosporidiosis, however, it still remains a major challenge in HIV/AIDS clinical management due to late diagnosis [32]. The delay in diagnosis and treatment of cryptosporidiosis arises due to multiple causative agents of chronic diarrhea in HIV infections [33]. Growing number of studies have reported cryptosporidiosis in HIV infected patients in Ghana [8, 28], but little or no effort has been done to identify the risk factors for transmission of *Cryptosporidium* infection in this group of patients. Poor personal hygiene, lack of sanitation facilities, sexual behavior patterns, closeness to domestic animal and drinking water have been reported as risk factors associated with cryptosporidiosis [14, 15]. Adjei *et al* could not establish any significant association between the risk factors and *Cryptosporidium* infection among HIV infected patients in Ghana [8]. In this study, we report the risk factors for *Cryptosporidium*, *Cyclospora* and *Microsporidium* infections in HIV/AIDS patients visiting HIV referral clinic in the Central region, Ghana.

The study showed that Cryptosporidiosis was the major diarrhea condition followed by cyclosporiasis in HIV/AIDS infected individuals. However, most of the cases of *Cyclospora* infections were frequently seen in a mix-infection with either *Cryptosporidium* or multiple infections of *Cryptosporidium*, *Cyclospora* and *Microsporidium*. The coinfection of *Cryptosporidium* and *Cyclospora* is increasingly being reported especially among HIV infected individuals [34, 35]. Previous reports have revealed that cyclosporiasis has a similar rate of complications in both immunocompromised and immunocompetent individuals [36]. Despite the small number of male subjects enrolled in the study, higher prevalences of infection in all categories from coinfections to single infections compared to the female counterparts were observed. A larger percentage of female subjects had no protozoan infections compared to the male subjects. There are several possible explanations to this finding, among them are the low levels of personal hygiene [37], less likely to seek medical care and may only do so when their clinical condition deteriorate [38] may account for their frequent contact to these opportunistic pathogens compared to their female counterpart. However, a larger sample size is required to confirm this observation.

We did not observe any single microsporidiosis, a diarrhea condition cause by only *Microsporidium* infections but rather was always seen as coinfection with other pathogens in HIV/AIDS patients enrolled in this study. Microsporidiosis, an infection caused by *Encephalitozoon* species in HIV/AIDS infection has been associated with clinical symptoms such as keratoconjunctivitis, peritonitis, encephalitis, myositis and nephritis but more frequently chronic diarrhea and malabsorption resulting in reduced serum concentration of D-xylose after ingestion of mannitol [39, 40]. In this report, *Microsporidium* infection was always seen in coinfection with *Cyclospora* or with *Cryptosporidium* and *Cyclospora* infections. This observation suggests that *Microsporidium* infections may not be a major diarrhoea-causing pathogen among our HIV/AIDS infected subjects, although it has been frequently reported in several studies conducted in HIV/AIDS infected individuals [41, 42]. Coinfection or multiple infections of *Microsporidium* with *Cryptosporidium* and *Cyclospora* infections may, however, act synergistically to invigorate the clinical conditions caused by *Cryptosporidium* or *Cyclospora* infections since these pathogens share similar disease-causing mechanisms [33].

CD4^+^ cells count lower than 200cells/mm^3^ have been associated with both cryptosporidiosis and cyclosporiasis [28]. Individually, each of the opportunistic parasites has been shown to be associated with depleted CD4^+^ cells in immunocompromised patients [43]. Several other studies have also reported coinfections of these opportunistic protozoan parasites in HIV/AIDS infected individuals with lower CD4^+^ cells count [44, 45].

In this study, we showed the prevalence of single and co-infection of *Cryptosporidium, Cyclospora & Microsporidium* with stratified CD4^+^ cells count. Although all groups of protozoan infections were observed among patients with CD4^+^ cell count lower than 200 or greater than 500 cells/mm^3^ with exception of *Cyclospora* & *Cryptosporidium* coinfections, it is not clear if this coinfection is protective among patients with CD4^+^ cell count <200 cells/mm3 and CD4+ cell count >500 cells/mm^3^. Nsagha *et al* [46], observed a similar phenomenon among HIV patients who were yet to be put on antiretroviral drugs. They recorded zero (0%) prevalence of *C. cayetenensis* among patients with CD4+ count <200 cell/mm^3^ and >500 cells/mm^3^. However, they observed 9.5% prevalence of *C. cayetenensis* among patients with CD4^+^ cells count between 200-499 cells/mm^3^. They also observed *C. cayetenensis* infection in all CD4^+^ cells count among patients who were already on antiretroviral drugs. Contrarily, Mitra *et al* [47] observed *Cyclospora spp* only in HIV-seropositive cases with CD4^+^ cells count <350 cells/μl but not in CD4^+^ cells count between 350-500 cells/μl or >500 cells/μl. Again, *Cyclospora spp* was only observed in acute diarrhea but not in chronic diarrhea or no diarrhea cases. Combining the observations made in this study and the previous two reports, the zero (0%) prevalence of *Cyclospora* & *Cryptosporidium* coinfections in CD4^+^ cells count <200 cells/mm^3^ and >500 cells/mm^3^ may not be due to chance but rather a phenomenon which requires further studies to understand.

In this study, we also delineated the social and behavioral risk factors associated with *Cryptosporidium, Cyclospora & Microsporidium* infections among the study subjects. Pearson chi-square statistics showed that *Cryptosporidium* infection is associated with drinking water. *Cyclospora* infection is also associated with the source of drinking water and the kind toilet of facilities used by the study subjects whilst *Microsporidium* showed association with the frequency of hand washing habits among the subjects. A more robust multivariate-adjusted statistics confirmed the source of drinking water as a risk factor for *Cryptosporidium* and *Cyclospora* infections among the HIV/AIDS patients attending the HIV referral clinic at Cape Coast.

The high occurrence of *Cryptosporidium* oocysts contamination in natural water sources coupled with its ability to withstand chlorine oxide, Alum-lime, chloramines and other treatment processes such as sedimentation, filtration or flocculation should make it a public health concern [17, 48, 49]. *Cyclospora* oocyst is also resistant to water treatment processes, however, shedding of oocysts by infected individuals including immunocompromised hosts is in very low numbers compared to *Cryptosporidium* [50]. Cryptosporidiosis is more aggressive in HIV/AIDS infections which requires meticulous efforts to reduce cryptosporidial associated morbidity and mortality.

In Ghana, contamination of water for irrigation, vegetable on sale at open-market, and plastic packaged drinking water “sachet water” by oocysts of *Cryptosporidium*, *Cyclospora* and other parasitic pathogens have been reported [49, 51]. Multivariate analysis showed significant differences between HIV/AIDS patients with “sachet water” and those with pipe-borne water as the source of drinking water in both *Cryptosporidium* and *Cyclospora* infections. A closer look at the prevalence showed that 76.2% of the subjects who use pipe-borne water as their source of drinking water had *Cryptosporidium* infections compared to 25% of the subjects with sachet water as their source of drinking water. This indicates that the sources of drinking water in Ghana is heavily contaminated with parasitic pathogens due to poor environmental sanitation [52]. Extra treatment systems such as UV water disinfection installation is required to complement the water treatment systems already in place in Ghana to improve potable water supply in the country [53]. Until such installations are put in place to improve the quality of water in Ghana, it is advisable especially for HIV/AIDS infected individuals to boil their drinking water to minimize infections of opportunistic pathogens. Boiling water inactivate the oocysts of *Cryptosporidium*, & *Cyclospora* and other infectious pathogens making it safe for drinking [54, 55].

In the case of *Microsporidium* infection, the possible risk factor for infection among the HIV/AIDS patients was identified in this study to be hand-washing behavior. Therefore, proper personal hygiene, improved sanitation and an extra treatment of drinking water would significantly reduce the exposure and infection of *Microsporidium* and other opportunistic pathogens [35].

## CONCLUSION

In conclusion, we report the risk factors for exposure of *Cryptosporidium*, *Cyclospora* and *Microsporidium* in HIV/AIDS infected individuals in Ghana for the first time. These findings should be of public health concern and measures should be put in place to improve the environmental sanitation and the quality of drinking water in Ghana. Clinicians should regularly offer advice to their HIV infected patients on how to improve their personal hygiene, their food source and an extra treatment of their drinking water to reduce opportunistic pathogenic infections.

## Figures and Tables

**Table 1:** Socio-demographic characteristics and its associated risks with opportunistic pathogens in HIV infected patients.

-	***Cyclospora spp***			***Cryptosporidium spp***		-	***Microsporidium spp***		-
	**Pos (%)**	**Neg (%)**	**X^2^**	**Pos (%)**	**Neg (%)**	**X^2^**	**Pos (%)**	**Neg (%)**	**X^2^**
**Gender**									
Male	6 (42.9)	8 (57.1)		8 (57.1)	6 (42.9)		4 (28.6)	10(71.4)	
Female	10(27.8)	26(72.2)	1.053^NS^	15(41.7)	21(58.3)	0.972 ^NS^	4 (11.1)	32(88.9)	2.286 ^NS^
**Age (years)**									
0-9	2 (50.0)	2 (50.0)		3 (75.0)	1 (25.0)		0 (0.0)	4(100.0)	
10-19	0 (0.0)	1(100.0)		0 (0.0)	1(100.0)		0 (0.0)	1(100.0)	
20-29	1 (20.0)	4 (80.0)		1 (20.0)	4 (80.0)		0 (0.0)	5(100.0)	
30-39	3 (25.0)	9 (75.0)		6 (50.0)	6 (50.0)		1 (8.3)	11(91.7)	
40-49	8 (44.4)	10(55.6)		8 (44.4)	10(55.6)		5 (27.8)	13(72.2)	
50-59	2 (33.3)	4 (66.7)		3 (50.0)	3 (50.0)		2 (33.3)	4 (66.7)	
>60	0 (0.0)	4(100.0)	4.836 ^NS^	2 (50.0)	2 (50.0)	3.726 ^NS^	0 (0.0)	4(100.0)	6.391 ^NS^
**Occupation**									
Civil servant	1(100.0)	0 (0.0)		0 (0.0)	1(100.0)		0 (0.0)	1(100.0)	
Trader	5(31.2)	11(68.8)		6 (37.5)	10(62.5)		3 (18.7)	13(81.3)	
Farmer	2 (40.0)	3 (60.0)		2 (40.0)	3 (60.0)		2 (40.0)	3 (60.0)	
Artisan	4 (50.0)	4 (50.0)		4 (50.0)	4 (50.0)		2 (25.0)	6 (75.0)	
Unemployed	2 (11.8)	15(88.2)		9 (52.9)	8 (47.1)		1 (5.9)	16(94.1)	
Student	2 (66.7)	1 (33.3)	8.323 ^NS^	2 (66.7)	1 (33.3)	2.287 ^NS^	0 (0.0%)	3(100.0)	4.772 ^NS^
**Residence**									
Within Cape Coast	11(32.4)	23(67.6)		18(52.9)	16(47.1)		6 (17.6)	28(82.4)	
Outside Cape Coast	5(31.3)	11(68.7)	0.006 ^NS^	5 (31.3)	11(68.7)	2.061 ^NS^	2 (12.5)	14(87.5)	0.214 ^NS^

NS significant (p>0.05); * Significant (p<0.05); ** Significant (p<0.01); *** Significant (p<0.001)

**Table 2:** Behavioral risk factors and its association with opportunistic pathogens in HIV infected patients.

-	***Cyclospora spp***			***Cryptosporidium spp***			***Microsporidium spp***		
-	**Pos (%)**	**Neg (%)**	**X^2^**	**Pos (%)**	**Neg (%)**	**X^2^**	**Pos (%)**	**Neg (%)**	**X^2^**
**Drinking water**									
Pipe borne water	13(61.9)	8(38.1)		16(76.2)	5(23.8)		6(28.6)	15(71.4)	
Sachet water	3(10.7)	25(89.3)		7(25.0)	21(75.0)		2 (7.1)	26(92.9)	
Bore hole water	0 (0.0)	1(100.0)	14.931***	0 (0.0)	1(100.0)	13.528***	0 (0.0)	1(100.0)	4.294 ^NS^
**Hand washing**									
Always	9(31.0)	20(69.0)		14(48.3)	15(51.7)		4(13.8)	25(86.2)	
Sometimes	3(25.0)	9(75.0)		6(50.0)	6(50.0)		0 (0.0)	12(100.0)	
Never/rarely	4 (44.4)	5(55.6)	0.923 ^NS^	3(33.3)	6(66.7)	0.719 ^NS^	4(44.4)	5 (55.6)	7.809*
**Toilet facility**									
Water closet (personal)	4 (36.4)	7 (63.6)		6 (54.5)	5 (45.5)		2 (18.2)	9 (81.8)	
Public toilet	11 (34.4%)	21 (65.6%)		14 (43.75%)	18 (56.25%)		6 (18.75%)	26 (81.25%)	
Household toilet	0 (0.0)	2 (100.0)		0 (0.0)	2 (100.0)		0 (0.0)	2 (100.0)	
Open defecation	1 (20.0)	4 (80.0)	12.463**	3 (60.0)	2 (40.0)	2.487 ^NS^	0 (0.0)	5 (100.0)	1.552 ^NS^
**Fruit washing**									
Always	7 (35.0)	13 (65.0)		11 (55.0)	9 (45.0)		5 (25.0)	15 (75.0)	
Sometimes	7 (30.4)	16 (69.6)		10 (43.5)	13 (56.5)		1 (4.3)	22 (95.7)	
Never/rarely	2 (28.6)	5 (71.4)	0.146 ^NS^	2 (28.6)	5 (71.4)	1.567 ^NS^	2 (28.6)	5 (71.4)	4.352 ^NS^

NS significant (p>0.05); * Significant (p<0.05); ** Significant (p<0.01); *** Significant (p<0.001)

**Table 3:** Prevalence of single and coinfections of protozoan pathogens among male and female HIV infected individuals.

-	**Male**	**Female**
**Triple infection**	2 (14.3%)	3 (8.3%)
**Double infection**		
*Microsporidium spp* & *Cyclospora spp*	2 (14.3%)	1 (2.8%)
*Cyclospora spp* & *Cryptosporidum spp*	1 (7.1%)	2 (5.6%)
**Single infection**		
*Cyclospora spp*	1 (7.1%)	1 (2.8%)
*Cryptosporidum spp*	5 (35.7%)	10 (27.8%)
No infection	3 (21.4%)	19 (52.8%)

**Table 4:** Distribution of single and coinfections of protozoan pathogens among HIV infected individuals with respect to CD4 ^+^ cells counts.

-	**CD4+ T cell count (cells/mm3)**		
	**<200**	**200-499**	**>500**
**Triple infection**	2 (12.5%)	1 (4.5%)	2 (16.7%)
**Double infection**			
*Microsporidium spp* & *Cyclospora spp*	1 (6.3%)	1 (4.5%)	1 (8.3%)
*Cyclospora spp* & *Cryptosporidium spp*	0 (0.0%)	3 (13.6%)	0 (0.0%)
**Single infection**			
*Cyclospora spp*	1 (6.3%)	0 (0.0%)	1 (8.3%)
*Cryptosporidium spp*	7 (43.8%)	4 (18.2%)	4 (33.3%)
No infection	5 (31.3%)	13 (59.1%)	4 (33.3%)

**Table 5:** Multivariate-adjusted odds to test the strength of association between behavioral risk factors and protozoan pathogens among HIV infected individuals.

-	***Cyclospora spp***		***Cryptosporidium spp***		***Microsporidium spp***	
	**OR (95% CI)**	**p**	**OR (95% CI)**	**P**	**OR (95% CI)**	**P**
**Drinking water**						
Pipe borne water	1.00¶ (referent)		1.00¶ (referent)		1.00¶ (referent)	
Sachet water	0.07 (0.02-0.33)	<0.001	0.10 (0.03-0.39)	<0.001	1.30 (0.27-6.20)	0.738
Bore hole water	0.02 (0.01-5.77)	0.356	0.11 (0.00-3.14)	0.198	1.76 (0.06-52.71)	0.744
**Hand washing**						
Always	1.00£ (referent)		1.00£ (referent)		1.00£ (referent)	
Sometimes	0.74 (0.16-3.40)	0.700	1.07 (0.28-4.12)	0.920	4.41 (0.22-88.54)	0.332
Never/rarely	1.78 (0.38-8.23)	0.462	0.54 (0.11-2.56)	0.435	20.45 (0.93-449.12)	0.050
**Toilet facility**						
Water closet (personal)	1.00λ (referent)		1.00λ (referent)		1.00λ (referent)	
Public toilet	0.92 (0.22-3.83)	0.905	0.17 (0.01-4.33)	0.283	0.76 (0.03-21.46)	0.872
Household toilet	0.33 (0.01-8.63)	0.508	0.65 (0.16-2.57)	0.537	1.04 (0.18-6.10)	0.967
Open defecation	0.44 (0.04-5.40)	0.512	1.25 (0.15-10.70)	0.839	6.75 (0.64-71.18)	0.112
**Fruit washing**						
Always	1.00ψ (referent)		1.00ψ (referent)		1.00ψ (referent)	
Sometimes	0.81 (0.23-2.92)	0.750	0.63 (0.19-2.10)	0.452	0.60 (0.12-3.08)	0.540
Never/rarely	0.74 (0.11-4.87)	0.757	0.33 (0.05-2.11)	0.240	0.67 (0.06-7.23)	0.739

¶ Adjusted variables other than source of drinking water ψ Adjusted variables other than fruit washing λ Adjusted variables other than toilet facility £ Adjusted variables other than hand washing
